# Global, regional, and national lifetime probabilities of urinary tract infections and interstitial nephritis from 1990 to 2021

**DOI:** 10.1186/s41043-025-00950-y

**Published:** 2025-07-03

**Authors:** Lingfeng Li, Yuhao Li, Yongming Chen, Huiming Hou, Jianye Wang, Ming Liu, Xin Wang, Shengfeng Wang

**Affiliations:** 1 Beijing Hospital, National Center of Gerontology, Institute of Geriatric Medicine, Chinese Academy of Medical Sciences & Peking Union Medical College, Beijing, China; 2 Department of Urology, Beijing Hospital, National Center of Gerontology, Institute of Geriatric Medicine, Beijing, China; 3 Department of Epidemiology and Biostatistics, School of Public Health, Peking University, Beijing, 100191, China; 4 Key Laboratory of Epidemiology of Major Diseases, Ministry of Education, Beijing, 100191, China; 5 Institute for Artificial Intelligence, Peking University, Beijing, China

**Keywords:** Urinary tract infection, Interstitial nephritis, Health inequity

## Abstract

Urinary tract infections (UTI) and interstitial nephritis (IN) are significant global health concerns, leaving a heavy burden to the healthcare system. UTI is one of the most common infections worldwide and IN is characterized by acute and chronic renal damage. This study aims to quantify the lifetime risk of developing and dying from UTI and IN across 204 countries from 1990 to 2021, utilizing data from the Global Burden of Disease 2021. We employed advanced statistical models, including the adjusted for multiple primaries method, Joinpoint trend analysis, and Autoregressive Integrated Moving Average (ARIMA) models, to assess temporal trends and forecast future risks. Results revealed a global lifetime risk of developing UTI and IN at 93.70% in 2021, with higher risks in females (96.05%) compared to males (77.27%). The global lifetime risk of dying from UTI and IN at 0.50%. High Socio-demographic Index (SDI) regions exhibited higher developing risks, while low-SDI regions showed significant upward trends. Gender disparities were evident, with females consistently experiencing higher risks. The ARIMA model projected a persistent upward trend in UTI and IN risks over the next 3 decades, emphasizing the need for targeted public health interventions. These findings highlight the importance of addressing UTI and IN through improved healthcare infrastructure, early screening, and preventive measures, particularly in high-risk populations and regions.

**Supplementary Information:**

The online version contains supplementary material available at 10.1186/s41043-025-00950-y.

## Introduction

UTI are one of the most common infections worldwide. UTI are mainly divided into upper urinary tract infections and lower urinary tract infections. Lower urinary tract infections cause lower urinary tract symptoms that affect the life quality, while upper urinary tract infections lead to systemic symptoms and even life-threatening situations. UTI threats the health of billions of people around the world, leaving a heavy burden to the healthcare system [1, 2]. UTI is prone to recurrent attacks and antibiotic resistance, making the disease more difficult to treat and exacerbating pelvic floor dysfunction in women [3, 4]. IN is a syndrome characterized by acute and chronic damage to the renal tubules and interstitium [5]. Medications and infections are the most common cause of IN. Medications are the leading cause in the developed countries, whereas infections are most common in undeveloped countries. Due to nonspecific symptoms of IN, a delay diagnosis usually happens, which lead to serious consequence such as kidney failure and heavier medical burden [6]. In 2021, the global lifetime risk of UTI and IN indicated nine in ten people developing and one in 200 people, which was higher in female [7]. Considering the huge magnitude of cases in UTI and IN, the disease-specific mortality cannot be ignored. During the past 30 years, epidemics of UTI and IN was not fully controlled so there was an urge need for sanitary system to strengthen disease prevention, screening, control and therapy according to the district, sexual, age and socioeconomic difference.

The concept of Lifetime Risk represents the cumulative probability of an individual developing a particular medical condition or health disorder throughout their lifespan. This epidemiological metric quantifies the overall chance of encountering specific health-related events from birth to death. As a crucial indicator in population health studies, it serves multiple essential functions, including evaluating health status, informing public health interventions, tracking disease prevalence patterns, and supporting clinical management decisions. Limited research has comprehensively evaluated the aggregated impact of urinary UTI and IN through the lens of lifetime risk analysis, particularly regarding condition-specific morbidity and fatality rates. Addressing this knowledge gap, our study aims to quantify both lifetime and age-specific probabilities of UTI and IN-related morbidity and mortality across 204 nations. Utilizing the Global Cancer Observatory (GLOBOCAN) 2021 dataset, a multi-level analysis encompassing global, regional, and national perspectives was conducted to provide a comprehensive assessment of disease burden. The objective of this research seek to inform national prevention and control strategies tailored to the cancer-specific lifetime risks of UTI and IN within world regions, ultimately to reduce the burden within existing resources and systems worldwide.

## Methods

### Data sources

Anonymized data from GBD 2021, a detailed database evaluating the incidence, point prevalence (hereafter referred to as prevalence), YLDs, YLLs, and DALYs for of 371 diseases, 88 risk factors, and injuries across 5 SDI categories and 204 countries and territories, was used in the study [7, 8]. Its extensive data sources encompass vital registration systems, epidemiological surveys, disease surveillance networks, cancer registries, law enforcement records, and open-access databases. Detailed methodologies for data collection and processing have been extensively documented in prior publications by GBD Collaborators in https://ghdx.healthdata.org/gbd-2021. Downstream data analysis employs advanced statistical modeling techniques, including meta-regression Bayesian, regularized, trimmed (MR-BRT) models, DisMod-MR 2.1, and spatiotemporal Gaussian process regression (ST-GPR). Standardization of disease nomenclature is achieved through the International Classification of Diseases (ICD) coding system, ensuring both accuracy and cross-comparability. The project adheres to the Guidelines for Accurate and Transparent Health Estimates Reporting (GATHER).

We extracted age-, sex-, and location-specific incidence and mortality rates for the UTI and IN, along with total population and all-cause mortality data, spanning the period from 1990 to 2021. The dataset encompasses all 21 GBD regions and 204 countries/territories (Table S6).

Furthermore, we incorporated the SDI, a composite metric that quantifies developmental status through the geometric mean of three key indicators: fertility rates among women under 25, educational attainment in adults aged 15 and above, and per capita income (https://ghdx.healthdata.org/record/global-burden-disease-study-2021-gbd-2021-socio-demographic-index-sdi-1950%E2%80%932021). These indicators are standardized on a scale ranging from 0 (lowest development level) to 1 (highest development level).

This study does not involve any individual-level or sensitive information and is exempt from ethical review requirements.

### Definition of UTI and IN

The GBD defines UTI as infections of the urinary system caused by bacterial, viral, or fungal pathogens, typically involving the bladder (lower urinary tract infection) or kidneys (upper urinary tract infection). Diagnosis of UTI is generally based on clinical symptoms (e.g., urinary frequency, urgency, and dysuria) and laboratory findings (e.g., urine culture or urinalysis).

IN is characterized in the GBD framework as a renal disorder marked by inflammation of the renal interstitium and tubular damage, often triggered by drug reactions, infections, or autoimmune conditions.

### Determination of lifetime risk

We employed the adjusted for multiple primaries method to estimate the lifetime risk of UTI and IN for the overall population, males, and females across 204 countries and territories globally [9, 10]. This approach accounts for competing risks of mortality from other causes and adjusts for the impact of multiple primary events included in incidence rates. Using age-specific incidence, mortality, and all-cause mortality data stratified by 5-year age groups, we calculated the lifetime risk of developing and dying from UTI and IN for males and females within each age stratum. This metric represents the cumulative probability of developing or dying from UTI and IN starting from the initial age group. Detailed methodology is provided in the supplementary materials.

### Calculation of average annual percentage change

Temporal trends in lifetime risk were assessed using the Average Annual Percentage Change (AAPC) and its 95% confidence interval (CI), calculated using the Joinpoint Trend Analysis Software (Command Line Version 5.3.0). AAPC is a summary measure of the trend over a pre-specified fixed interval. It allows us to use a single number to describe the average APCs over a period of multiple years. It is valid even if the joinpoint model indicates that there were changes in trends during those years. It is computed as a weighted average of the APCs from the joinpoint model, with the weights equal to the length of the APC interval [11]. A negative AAPC indicates a declining trend, while a positive AAPC signifies an increasing trend.

### Autoregressive integrated moving average model

We utilized the ARIMA model to predict the lifetime risk of UTI and IN from 2019 to 2050. ARIMA model is a time series model widely used in analyzing and forecasting time series data. Model selection was based on autocorrelation and partial autocorrelation analyses to determine the orders of autoregressive (AR) and moving average (MA) components. To ensure optimal model complexity and predictive accuracy, we employed statistical criteria, including the Akaike Information Criterion (AIC), Bayesian Information Criterion (BIC), and Root Mean Square Error (RMSE), for model optimization [12, 13]. Detailed methodology is provided in the supplementary materials.

### Gender differences

Assuming a uniform distribution of lifetime risk within the given 95% CI, we calculated the male-to-female lifetime risk ratio and its 95% CI through 100,000 resampling iterations.

### Concentration index

Concentration Index was employed to quantify the inequality in burden of UTI and IN based on the Socio-demographic Index (SDI) across countries with varying development levels. The Concentration Index ranges from − 1 to 1, where values less than 0 indicate that the disease burden is concentrated in low-SDI regions, and values greater than 0 suggest a concentration in high-SDI regions [14]. Detailed methodology is provided in the supplementary materials.

### Frontier analysis

To evaluate the relationship between disease burden and socio-demographic development, we applied Frontier Analysis as a quantitative method to determine the achievable lifetime risk based on development levels measured by SDI. Detailed methodology is provided in the supplementary materials.

### Statistical analysis

The 95% CI was calculated by assuming a Poisson distribution for disease incidence and mortality rates. *P* value less than 0.05 in both sides was considered as significant. Citation program used is NoteExpress (Version 4.2.0.10156). All analyses were conducted with SAS (Version 9.4) and R software (Version 4.4.1).

## Results

### Lifetime risk of UTI and IN in different regions and gender in 2021

**Fig. 1:**
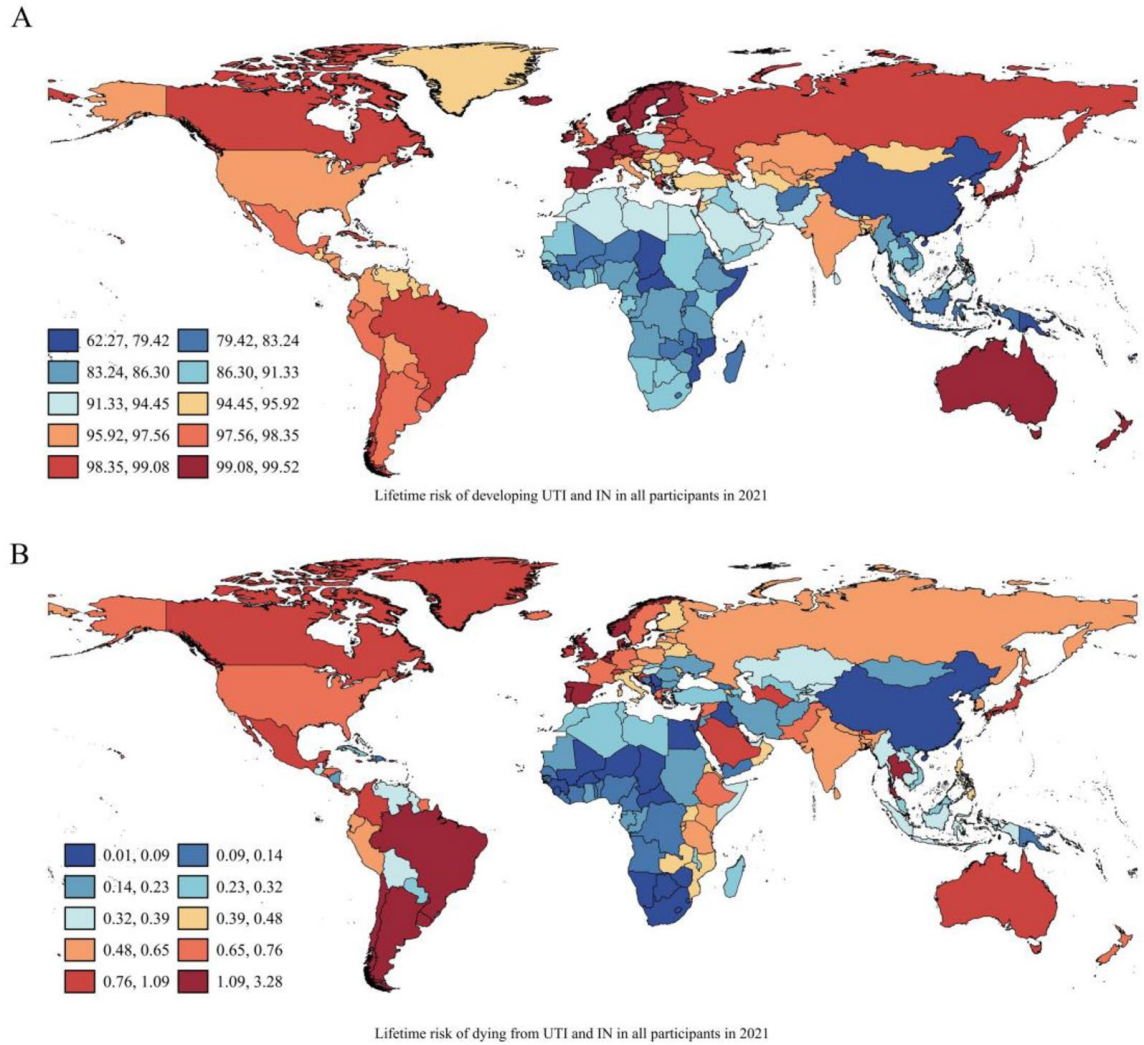
Lifetime risk of developing and dying from UTI and IN across 204 countries in 2021 globally. **A** Global lifetime risk of developing UTI and IN in all participants in 2021. **B** Global lifetime risk of dying from UTI and IN in all participants in 2021

In 2021, the estimated global lifetime risk (from birth to death) of developing UTI and IN was 93.70% (95% CI 93.69–93.71), 77.27% (95% CI 77.24–77.30) in male and 96.05% (95% CI 96.04–96.07) in female respectively (Table 1). The estimated global lifetime risk of dying from UTI and IN was 0.50% (95% CI 0.50–0.50), 0.41% (95% CI 0.40–0.41) in male and 0.60% (95% CI 0.60–0.61) in female (Table S1). In general, the lifetime risk of developing and dying from UTI and IN were higher in female than male. Among all the participants, lifetime risk of developing UTI and IN was highest in Australasia, following by Western Europe and Eastern Europe, and lowest in East Asia. In the male population, lifetime risk of developing UTI and IN was highest in Australasia, following by the Tropical Latin America and South Asia, and lowest in East Asia. Among the female population, lifetime risk of developing UTI and IN was highest in High-income Asia Pacific, following by the Australasia and Western Europe, and lowest in East Asia (Table 1, Fig. 1). Regarding lifetime risk of dying from UTI and IN, Tropical Latin America (sex combined: 1.97% CI 1.95–1.99, female: 2.55% CI 2.51–2.59, male 1.44% CI 1.41–1.46) and Southern Latin America (sex combined: 1.64% CI 1.60–1.67, female 2.03% CI 1.98–2.09, male 1.24% CI 1.20–1.29) rank first and second respectively (Table S1). Southern Sub-Saharan Africa exhibited lowest lifetime dying risk (sex combined: 0.07% CI 0.06–0.07, female 0.08% CI 0.07–0.09, male 0.06% CI 0.05–0.06) among all these regions in 2021.

**Table 1:** Lifetime risks of developing UTI and IN in 2021

Regions	Birth to death	Age 40 to death	Age 50 to death	Age 60 to death	Age 70 to death	Age 80 to death
Global	93.70(93.69–93.71)	11.01(11.00–11.01)	4.38(4.38–4.38)	1.63(1.63–1.63)	0.53(0.53–0.53)	0.13(0.13–0.13)
High-income North America	97.53(97.49–97.57)	11.92(11.91–11.93)	5.61(5.61–5.62)	2.33(2.32–2.33)	0.70(0.69–0.70)	0.09(0.09–0.09)
Caribbean	94.52(94.36–94.67)	4.21(4.21–4.22)	1.12(1.12–1.12)	0.30(0.29–0.30)	0.08(0.08–0.08)	0.02(0.02–0.02)
Andean Latin America	97.84(97.75–97.94)	0.84(0.84–0.84)	0.12(0.12–0.12)	0.02(0.02–0.02)	0.00(0.00–0.00)	0.00(0.00–0.00)
Central Latin America	97.49(97.46–97.52)	5.92(5.91–5.92)	1.85(1.85–1.85)	0.45(0.45–0.45)	0.11(0.11–0.11)	0.02(0.02–0.02)
Tropical Latin America	98.40(98.36–98.45)	0.53(0.53–0.53)	0.09(0.09–0.09)	0.02(0.02–0.02)	0.00(0.00–0.00)	0.00(0.00–0.00)
North Africa and Middle East	91.49(91.45–91.53)	12.05(12.04–12.06)	5.38(5.38–5.39)	2.12(2.12–2.13)	0.72(0.72–0.72)	0.16(0.16–0.16)
South Asia	95.45(95.43–95.48)	4.02(4.02–4.02)	0.91(0.91–0.91)	0.17(0.17–0.17)	0.03(0.03–0.03)	0.00(0.00–0.00)
Central Sub-Saharan Africa	83.47(83.35–83.58)	17.73(17.69–17.76)	7.20(7.18–7.22)	2.24(2.23–2.25)	0.54(0.53–0.54)	0.06(0.06–0.07)
Eastern Sub-Saharan Africa	82.64(82.57–82.71)	20.27(20.25–20.30)	8.79(8.77–8.80)	2.75(2.74–2.76)	0.65(0.64–0.65)	0.08(0.08–0.08)
Southern Sub-Saharan Africa	87.93(87.78–88.08)	9.77(9.75–9.78)	3.25(3.24–3.26)	0.84(0.84–0.84)	0.18(0.18–0.18)	0.03(0.03–0.03)
Western Sub-Saharan Africa	84.29(84.24–84.34)	15.59(15.58–15.60)	5.96(5.96–5.97)	1.85(1.85–1.86)	0.50(0.49–0.50)	0.07(0.07–0.07)
Oceania	71.09(70.51–71.67)	30.87(30.62–31.12)	15.10(14.93–15.27)	5.88(5.78–5.97)	1.71(1.67–1.75)	0.31(0.29–0.33)
Central Asia	96.26(96.19–96.34)	8.33(8.33–8.34)	3.01(3.01–3.02)	0.84(0.84–0.84)	0.17(0.17–0.17)	0.02(0.02–0.02)
Central Europe	95.00(94.94–95.05)	15.38(15.36–15.41)	9.90(9.88–9.92)	5.23(5.22–5.24)	1.93(1.93–1.93)	0.37(0.37–0.37)
High-middle SDI	94.15(94.13–94.18)	17.07(17.06–17.07)	8.81(8.81–8.81)	4.09(4.08–4.09)	1.42(1.42–1.42)	0.36(0.36–0.36)
High SDI	98.23(98.21–98.25)	8.72(8.71–8.72)	4.05(4.04–4.05)	1.80(1.80–1.80)	0.73(0.73–0.73)	0.19(0.19–0.19)
Low-middle SDI	94.28(94.26–94.30)	6.85(6.85–6.85)	2.00(2.00–2.00)	0.49(0.49–0.49)	0.10(0.10–0.10)	0.01(0.01–0.01)
Low SDI	87.91(87.88–87.94)	12.83(12.82–12.83)	4.55(4.55–4.55)	1.28(1.28–1.29)	0.28(0.28–0.28)	0.03(0.03–0.03)
Middle SDI	95.16(95.13–95.18)	12.64(12.64–12.64)	5.00(5.00–5.00)	1.83(1.83–1.83)	0.55(0.55–0.55)	0.13(0.13–0.13)
East Asia	64.33(64.26–64.41)	35.48(35.44–35.51)	22.48(22.46–22.51)	11.52(11.51–11.54)	4.59(4.58–4.59)	1.20(1.20–1.20)
Eastern Europe	98.93(98.91–98.95)	2.86(2.86–2.86)	1.03(1.02–1.03)	0.25(0.25–0.25)	0.04(0.04–0.04)	0.00(0.00–0.00)
High-income Asia Pacific	98.90(98.88–98.93)	7.15(7.14–7.15)	3.51(3.51–3.51)	1.65(1.65–1.65)	0.83(0.83–0.83)	0.33(0.33–0.33)
Australasia	99.37(99.28–99.45)	2.02(2.02–2.02)	0.66(0.66–0.66)	0.24(0.24–0.24)	0.09(0.09–0.09)	0.03(0.03–0.03)
Western Europe	99.04(99.00–99.09)	3.47(3.47–3.47)	1.20(1.19–1.20)	0.44(0.44–0.44)	0.17(0.17–0.17)	0.06(0.06–0.06)
Southeast Asia	85.81(85.73–85.88)	34.24(34.22–34.26)	16.99(16.98–17.00)	6.32(6.32–6.33)	1.85(1.84–1.85)	0.38(0.38–0.38)
Southern Latin America	98.10(97.98–98.22)	6.25(6.25–6.26)	2.35(2.34–2.35)	0.83(0.83–0.83)	0.24(0.24–0.24)	0.05(0.05–0.05)
*Male*						
Global	77.27(77.24–77.30)	25.55(25.54–25.57)	14.69(14.69–14.70)	7.86(7.86–7.87)	3.40(3.39–3.40)	0.99(0.99–0.99)
High-income North America	77.42(77.21–77.62)	45.95(45.85–46.06)	36.87(36.80–36.94)	26.10(26.06–26.14)	13.81(13.79–13.83)	3.64(3.63–3.64)
Caribbean	78.79(78.41–79.17)	26.46(26.33–26.60)	14.24(14.15–14.33)	6.59(6.54–6.64)	2.37(2.35–2.39)	0.55(0.54–0.56)
Andean Latin America	89.23(88.98–89.48)	22.42(22.36–22.49)	10.28(10.23–10.32)	4.26(4.24–4.28)	1.37(1.36–1.38)	0.24(0.24–0.24)
Central Latin America	81.33(81.18–81.49)	26.89(26.83–26.96)	15.13(15.09–15.18)	7.57(7.54–7.59)	3.05(3.04–3.06)	0.71(0.71–0.72)
Tropical Latin America	92.13(92.00–92.26)	21.38(21.36–21.41)	9.89(9.87–9.90)	4.04(4.03–4.05)	1.18(1.18–1.18)	0.19(0.19–0.19)
North Africa and Middle East	69.20(69.09–69.31)	21.11(21.06–21.16)	11.00(10.96–11.04)	4.81(4.79–4.83)	1.63(1.62–1.64)	0.38(0.38–0.39)
South Asia	90.17(90.13–90.20)	16.44(16.43–16.44)	6.64(6.63–6.64)	2.32(2.31–2.32)	0.61(0.61–0.61)	0.09(0.09–0.09)
Central Sub-Saharan Africa	67.68(67.45–67.91)	21.70(21.59–21.81)	10.61(10.54–10.67)	3.80(3.77–3.83)	1.00(0.99–1.01)	0.10(0.10–0.11)
Eastern Sub-Saharan Africa	64.05(63.91–64.19)	22.80(22.73–22.87)	11.65(11.60–11.69)	4.37(4.34–4.39)	1.17(1.16–1.18)	0.14(0.14–0.14)
Southern Sub-Saharan Africa	73.28(73.00–73.56)	15.34(15.26–15.41)	6.19(6.15–6.23)	1.82(1.80–1.84)	0.38(0.38–0.39)	0.05(0.04–0.05)
Western Sub-Saharan Africa	65.64(65.53–65.76)	19.12(19.05–19.18)	9.97(9.92–10.01)	4.23(4.21–4.25)	1.29(1.28–1.30)	0.21(0.21–0.22)
Oceania	38.32(37.29–39.35)	22.87(22.30–23.44)	15.19(14.81–15.57)	7.81(7.59–8.03)	2.49(2.38–2.60)	0.45(0.40–0.50)
Central Asia	83.84(83.63–84.05)	26.47(26.38–26.56)	14.16(14.10–14.21)	6.01(5.98–6.05)	1.74(1.72–1.75)	0.26(0.25–0.26)
Central Europe	79.98(79.80–80.16)	19.88(19.78–19.98)	15.33(15.26–15.40)	10.02(9.99–10.06)	4.56(4.54–4.58)	1.07(1.07–1.08)
High-middle SDI	69.77(69.70–69.85)	24.75(24.71–24.79)	16.60(16.57–16.63)	9.91(9.89–9.93)	4.18(4.17–4.19)	1.17(1.16–1.17)
High SDI	76.33(76.23–76.42)	37.82(37.77–37.88)	28.49(28.45–28.53)	19.77(19.75–19.80)	11.36(11.35–11.38)	4.14(4.13–4.15)
Low-middle SDI	84.83(84.78–84.88)	20.97(20.96–20.99)	9.64(9.63–9.65)	3.80(3.79–3.80)	1.12(1.12–1.13)	0.19(0.19–0.19)
Low SDI	74.40(74.33–74.47)	21.58(21.54–21.61)	10.47(10.45–10.49)	4.08(4.07–4.09)	1.13(1.12–1.13)	0.15(0.15–0.15)
Middle SDI	75.39(75.33–75.44)	27.58(27.56–27.61)	15.56(15.55–15.58)	8.09(8.08–8.11)	3.23(3.22–3.23)	0.85(0.85–0.86)
East Asia	24.43(24.30–24.57)	16.28(16.21–16.34)	11.94(11.90–11.98)	7.39(7.36–7.41)	3.50(3.48–3.51)	1.08(1.07–1.09)
Eastern Europe	95.82(95.78–95.86)	6.99(6.97–7.00)	3.64(3.63–3.65)	1.39(1.39–1.39)	0.34(0.34–0.34)	0.04(0.04–0.04)
High-income Asia Pacific	86.67(86.57–86.77)	22.04(21.97–22.10)	15.48(15.42–15.53)	10.24(10.20–10.28)	6.13(6.11–6.15)	2.61(2.60–2.63)
Australasia	79.27(78.75–79.79)	42.39(42.10–42.68)	29.60(29.37–29.83)	17.86(17.69–18.02)	9.16(9.06–9.26)	3.36(3.31–3.40)
Western Europe	65.32(65.14–65.50)	37.97(37.88–38.07)	26.64(26.57–26.72)	16.26(16.21–16.31)	8.05(8.02–8.07)	2.85(2.84–2.86)
Southeast Asia	54.12(53.96–54.28)	32.35(32.28–32.43)	20.77(20.72–20.82)	10.16(10.13–10.19)	3.54(3.52–3.55)	0.80(0.79–0.80)
Southern Latin America	68.86(68.37–69.34)	41.79(41.52–42.05)	31.09(30.89–31.30)	19.50(19.38–19.63)	8.78(8.71–8.84)	2.33(2.30–2.36)
*Female*						
Global	96.05(96.04–96.07)	3.46(3.46–3.46)	0.92(0.92–0.92)	0.24(0.24–0.24)	0.06(0.06–0.06)	0.01(0.01–0.01)
High-income North America	99.21(99.17–99.25)	2.40(2.40–2.40)	0.69(0.69–0.69)	0.17(0.17–0.17)	0.03(0.03–0.03)	0.00(0.00–0.00)
Caribbean	96.03(95.86–96.20)	0.51(0.51–0.51)	0.07(0.07–0.07)	0.01(0.01–0.01)	0.00(0.00–0.00)	0.00(0.00–0.00)
Andean Latin America	98.42(98.33–98.52)	0.02(0.02–0.02)	0.00(0.00–0.00)	0.00(0.00–0.00)	0.00(0.00–0.00)	0.00(0.00–0.00)
Central Latin America	98.87(98.84–98.89)	1.00(1.00–1.00)	0.18(0.18–0.18)	0.02(0.02–0.02)	0.00(0.00–0.00)	0.00(0.00–0.00)
Tropical Latin America	98.95(98.91–98.99)	0.01(0.01–0.01)	0.00(0.00–0.00)	0.00(0.00–0.00)	0.00(0.00–0.00)	0.00(0.00–0.00)
North Africa and Middle East	96.77(96.72–96.81)	4.96(4.96–4.96)	1.81(1.81–1.81)	0.63(0.62–0.63)	0.20(0.20–0.20)	0.04(0.04–0.04)
South Asia	96.28(96.25–96.30)	0.80(0.80–0.80)	0.09(0.09–0.09)	0.01(0.01–0.01)	0.00(0.00–0.00)	0.00(0.00–0.00)
Central Sub-Saharan Africa	89.53(89.40–89.65)	11.97(11.95–11.99)	3.98(3.97–3.99)	1.06(1.06–1.07)	0.23(0.23–0.24)	0.03(0.03–0.03)
Eastern Sub-Saharan Africa	89.26(89.18–89.34)	14.44(14.43–14.45)	5.18(5.17–5.19)	1.35(1.34–1.35)	0.29(0.28–0.29)	0.04(0.03–0.04)
Southern Sub-Saharan Africa	92.71(92.54–92.89)	5.03(5.02–5.04)	1.32(1.32–1.32)	0.30(0.30–0.30)	0.06(0.06–0.06)	0.01(0.01–0.01)
Western Sub-Saharan Africa	88.75(88.70–88.81)	9.31(9.31–9.32)	2.56(2.56–2.56)	0.61(0.61–0.62)	0.15(0.15–0.15)	0.02(0.02–0.02)
Oceania	84.74(84.08–85.40)	27.08(26.91–27.24)	10.72(10.61–10.83)	3.46(3.40–3.52)	0.93(0.90–0.96)	0.17(0.16–0.18)
Central Asia	97.94(97.85–98.02)	2.07(2.07–2.07)	0.50(0.50–0.50)	0.10(0.10–0.10)	0.02(0.02–0.02)	0.00(0.00–0.00)
Central Europe	98.58(98.53–98.63)	8.65(8.64–8.66)	4.66(4.66–4.67)	2.02(2.02–2.02)	0.62(0.62–0.62)	0.10(0.10–0.10)
High-middle SDI	98.47(98.45–98.50)	7.47(7.47–7.48)	2.95(2.95–2.95)	1.09(1.09–1.09)	0.32(0.32–0.32)	0.07(0.07–0.07)
High SDI	99.45(99.43–99.47)	1.32(1.32–1.32)	0.37(0.37–0.37)	0.11(0.11–0.11)	0.03(0.03–0.03)	0.01(0.01–0.01)
Low-middle SDI	95.83(95.80–95.86)	1.82(1.82–1.82)	0.32(0.32–0.32)	0.05(0.05–0.05)	0.01(0.01–0.01)	0.00(0.00–0.00)
Low SDI	91.26(91.22–91.30)	6.21(6.20–6.21)	1.54(1.54–1.54)	0.31(0.31–0.31)	0.05(0.05–0.05)	0.01(0.01–0.01)
Middle SDI	97.86(97.84–97.88)	4.09(4.09–4.09)	1.09(1.09–1.09)	0.29(0.29–0.29)	0.07(0.07–0.07)	0.01(0.01–0.01)
East Asia	83.99(83.90–84.07)	37.73(37.70–37.75)	21.31(21.29–21.33)	9.75(9.74–9.77)	3.56(3.56–3.57)	0.88(0.88–0.89)
Eastern Europe	99.47(99.44–99.49)	0.96(0.96–0.96)	0.24(0.24–0.24)	0.04(0.04–0.04)	0.01(0.01–0.01)	0.00(0.00–0.00)
High-income Asia Pacific	99.74(99.72–99.76)	1.60(1.60–1.60)	0.53(0.53–0.54)	0.18(0.18–0.18)	0.07(0.07–0.08)	0.03(0.03–0.03)
Australasia	99.67(99.60–99.75)	0.07(0.07–0.07)	0.01(0.01–0.01)	0.00(0.00–0.00)	0.00(0.00–0.00)	0.00(0.00–0.00)
Western Europe	99.60(99.55–99.64)	0.18(0.18–0.18)	0.03(0.03–0.03)	0.01(0.01–0.01)	0.00(0.00–0.00)	0.00(0.00–0.00)
Southeast Asia	94.25(94.16–94.33)	23.86(23.85–23.87)	9.04(9.03–9.04)	2.60(2.60–2.60)	0.65(0.65–0.65)	0.12(0.12–0.12)
Southern Latin America	99.10(98.98–99.22)	0.63(0.63–0.63)	0.12(0.12–0.12)	0.03(0.03–0.03)	0.01(0.01–0.01)	0.00(0.00–0.00)

### Shifting in lifetime risk from 1990 to 2021

From 1990 to 2021, Australasia ranked first in lifetime risk of developing UTI and IN throughout the whole period, while East Asia had the lowest lifetime risk of developing UTI and IN (Fig. S1A). However, there were divergence among different genders. Among female, High-income Pacific Asia ranked first in lifetime risk of developing UTI and IN in most time from 1990 to 2021 (Fig. S1B). Among female, High-income Pacific Asia ranked first in lifetime risk of developing UTI and IN in most time from 1999 to 2021(Fig. S1C). Regarding the lifetime risk rate of dying from UTI and IN, there has been a significant change in ranking. High income North America ranked first in the period from 1990 to 1997, and decreased during 1998 to 2021. Ranking of lifetime risk rate of dying from UTI and IN Tropical Latin America and Southern Latin America rose between 1990 and 2021 and remained in the top two in the latter half of the period. The lifetime risk rate of dying from UTI and IN in Southern Sub-Saharan Africa and East Asia remained in the bottom two from 1990 to 2021 (Fig. S1D). In female population, Among the female population, the two regions with the lowest lifetime risk rate of dying from UTI and IN are Central Sub-Saharan Africa and Southern Sub-Saharan Africa, while the rest of the ranking trends are similar to the overall population. The trend of male population is similar to the overall population. To assess the trends over this period, we determined the average annual percent change (AAPC) for each region. Eastern Sub-Saharan Africa, Central Sub-Saharan Africa and Western Sub-Saharan Africa exhibited the top third average change in lifetime risk of developing UTI and IN (Fig. S2, Table S2). High-income North America showed the least increased trend in all participant and female, and Southern Sub-Saharan Africa in male respectively (Fig. S2, Table S2). In the ranking of dying risk, Southern Latin America displayed the most significant increase while Southern Sub-Saharan Africa showed a significant decrease compared with the other regions regardless of male of female (Fig. S3, Table S2).

### Socioeconomic inequality in UTI and IN

**Fig. 2:**
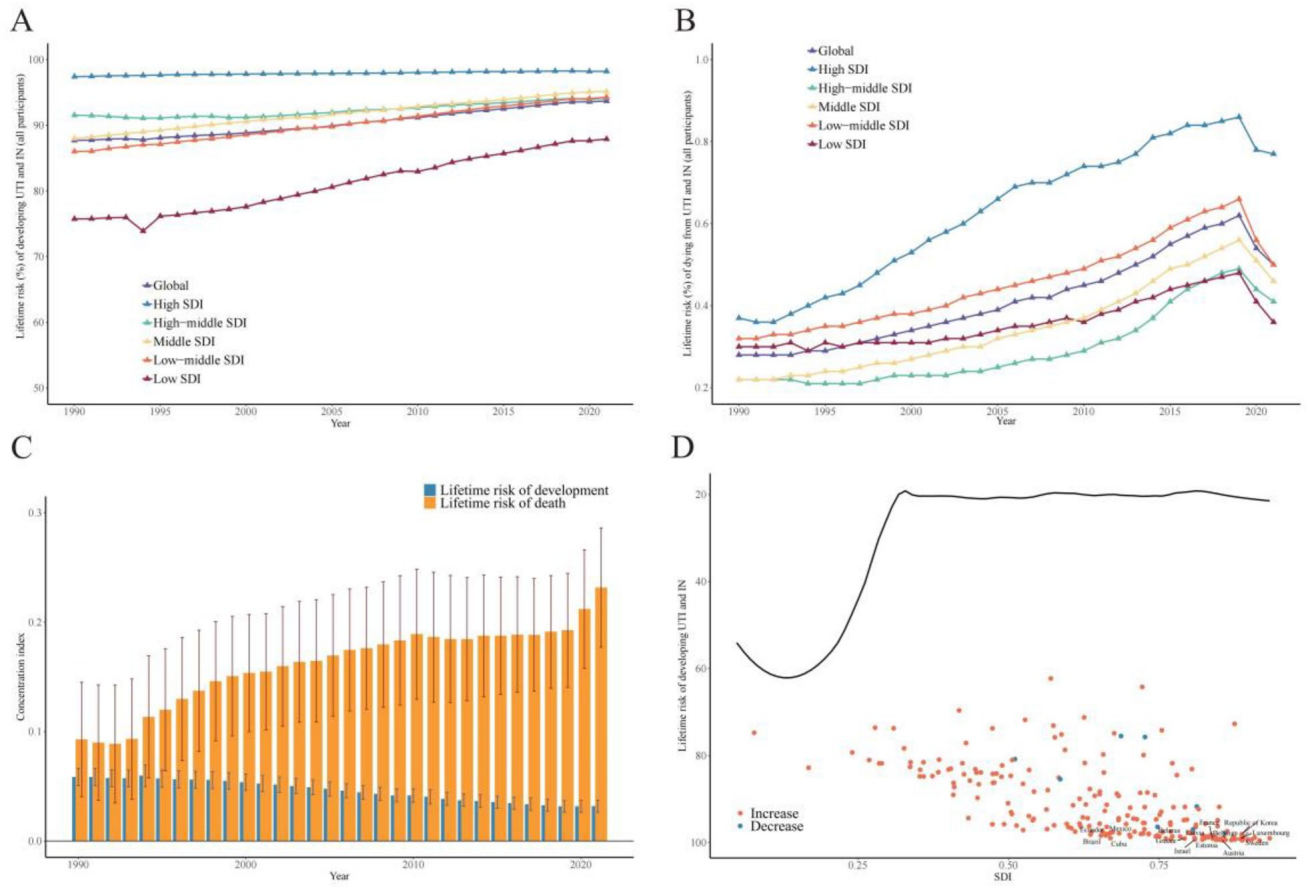
Relationship between socioeconomic inequality and lifetime risk of UTI and IN. **A** Developing risk in different SDI level from 1990 to 2021 globally. **B** Dying risk in different SDI level from 1990 to 2021 globally. **C** CI values of developing and dying lifetime risk from 1990 to 2021 globally. The CI reflects the disparity in disease burden among various SDI groups, where higher values suggest a more pronounced clustering of risk in areas with higher SDI. The error bars denote the 95% CI. **D** Frontier Analysis was utilized to quantify the disparity between observed lifetime risks (from birth to death) across nations and the theoretically attainable minimum risk for their respective SDI levels. The fitted model's frontier, depicted by the black curve, delineates the lowest achievable risk corresponding to each SDI value. Individual data points on the scatter plot correspond to the actual lifetime risk estimates for each country. Among these, the 15 nations exhibiting the most pronounced deviations from the frontier are highlighted in black

We further explore the relationship between different socio-economic factors and lifetime risks of UTI and IN. In all participants, developing risk in High SDI regions were higher than the low SDI regions, the trend is opposite to SDI overall (Table 1, Fig. 2A). All SDI levels showed an upward trend in developing risk during 1990 to 2021, and the upward trend is most evident in low SDI regions. Dying risk in high SDI regions was significantly higher than the other SDI regions and exhibited the most significant upward trend from 1990 to 2019 (Fig. 2B, Table S3). Until 2019 to 2021, dying risk in all SDI levels experienced an unexpected nosedive and low SDI regions ranked lowest among all SDI levels.

There was also deviation in lifetime risk from different SDI regions in different sex. The female population shared the same alteration pattern with all participants (Table 1, Fig. S2). In male population, low-middle SDI region ranked the most in developing risk from 1990 to 2021 (Fig. S2). The SDI levels showed no positive correlation with developing risk. In dying risk, SDI ranking and alteration pattern in male population was similar to the female population and all participant.

To further explore the socioeconomic inequality in the developing and dying risk of UTI and IN, we calculated the Concentration Index from 1990 to 2021. Concentration Index greater than 0 indicates that the disease burden is mainly concentrated in areas with higher SDI.

Both female and male population, Concentration Index of developing and dying risk of UTI and IN were consistently above 0 from 1990 to 2021, and showed an upward trend (Fig. 2C, Fig. S2). Moreover, Frontier Analysis indicated that there was a huge gap between achievable minimal lifetime developing risk and real risk across 204 regions, which is more obvious in higher SDI regions (Fig. 2D, Table S4).

### Life time risk alteration of UTI and IN across age

**Fig. 3:**
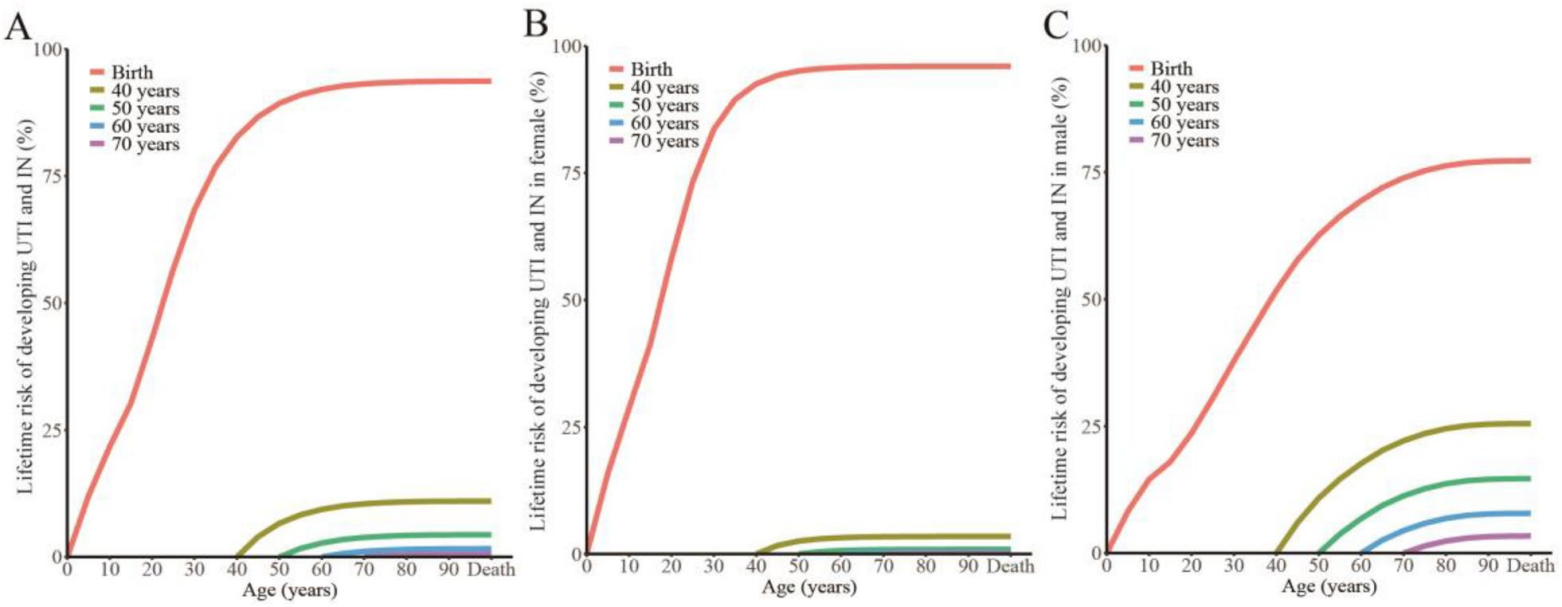
Lifetime risk of developing UTI and IN in different age groups. **A** The curves illustrate the likelihood of developing UTI and IN starting from an age at which an individual is free of the condition up to a defined age range. **B** and **C** represented the female and male participants respectively

In all participant, lifetime developing risk decline with increasing age (Table 1, Fig. 3 A). In female, thought the global lifetime developing risk higher than male, there was a sharp decline at age 40 (3.46%) and less than 1% after age 50. In male, except the risk from birth to death, the global lifetime developing risk from age 40, 50, 60, 70 and 80 all higher than female respectively. Until age 80, developing risk in male was less than 1% (0.99%).

The dying risk from UTI and IN varies slightly with age, representing a slow downward trend (Table S1). Dying risk in female was higher than male across all age groups. Both male and female possessed the highest dying risk at age 40 (male: 0.39%, female: 0.59%).

### Forecasting future global lifetime risk of UTI and IN

**Fig. 4:**
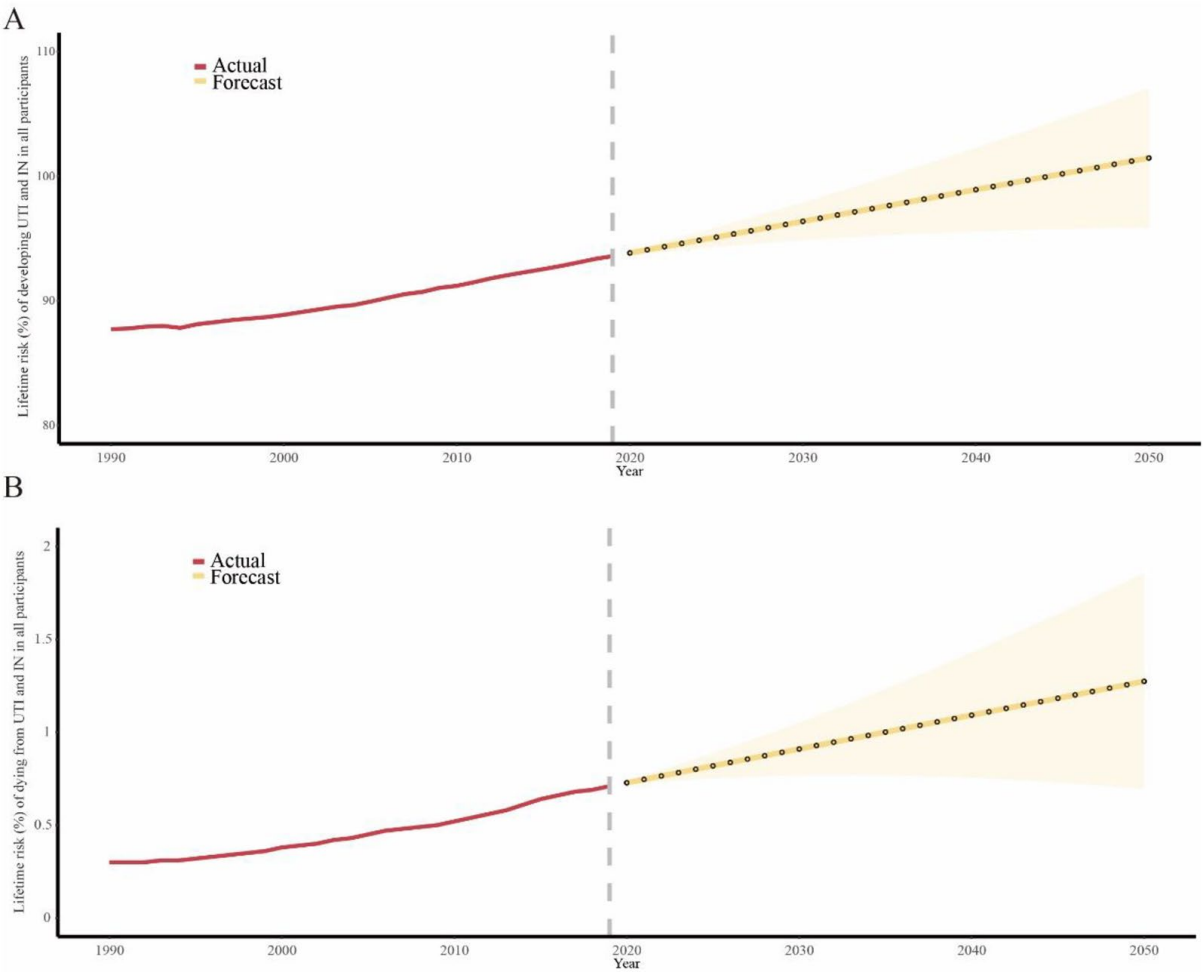
ARIMA algorithm forecast the lifetime risk of UTI and IN in the next 30 years. **A** and **B** The red curve represented the lifetime risk of developing (or dying risk) UTI and IN from 1990 to 2019 and the yellow curve forecasted the developing risk from 2020 to 2050. Yellow regions represented 95% CI for the forecasted risk

To forecast the global lifetime risk of developing and dying from urinary tract infections UTI and IN over the next 3 decades, we employed an ARIMA model. The results demonstrated a persistent upward trend in risk, building upon the gradual rise observed in previous decades (Fig. 4, Table S5). These outcomes offer crucial perspectives for addressing the worldwide impact of UTI and IN, informing the formulation of specific preventive measures.

## Discussion

Our study revealed a significant increase in the lifetime risk of UTI and IN over the past 4 decades, with marked regional disparities. The global burden of UTI and IN aligns with broader trends in infectious and chronic kidney diseases, as highlighted by recent Global Burden of Disease (GBD) studies [15, 16]. In 2021, the epidemiological landscape revealed a consistent upward trend in lifetime developing and dying risk. Globally, the lifetime developing risk exceeded 90% for females, 70% for male, with particularly higher risk observed in developed regions, indicating a phenomenon of high economic status correlating with elevated disease burden. This suggests a potential need for a shift in healthcare delivery models. Additionally, a notable gender disparity in disease distribution was observed. The anatomy of the female urethra makes it easy for bacteria to invade the urethra and cause inflammation. Older women and women with pelvic floor dysfunction are more likely to suffer from recurrent UTI and complicated UTI, making the disease more difficult to cure. Consequently, when developing management based on gender disparity in lifetime risk, it is important to incorporate the characteristics of different genders in terms of disease onset.

Regarding mortality, most regions reported rates below 1% in 2021, with the exception of Southern Latin America and Tropical Latin America, where rates exceeded 1% for both genders. This highlights the urgent need for targeted interventions to mitigate lifetime mortality in these regions.

From 1990 to 2021, the incidence of UTI and IN remained persistently high in regions with high SDI, indicating inadequate disease control over time. Although African regions ranked lower in global incidence rates by 2021, their AAPC in developing risk was among the highest, warranting close monitoring and intervention. Notably, all regions exhibited an AAPC greater than 0, reflecting a universal upward trend in UTI and IN incidence during this period.

In terms of lifetime mortality, most regions also showed an AAPC greater than 0, indicating an increasing trend. However, certain areas such as Oceania and Southern Sub-Saharan Africa demonstrated a decline in lifetime mortality, suggesting potential success in local disease management strategies.

The SDI level significantly influences the lifetime developing and dying risk of UTI and IN. Analysis of lifetime risk revealed the potential risk in higher SDI regions compared with the other statistical analysis methods in previous reports [16]. In high-SDI regions, where the risk of disease onset is elevated, it is recommended to promote early screening for UTI and IN, particularly among high-risk populations such as the elderly and individuals with chronic conditions [17]. Despite better healthcare infrastructure in these regions, antibiotic resistance UTI and medication induced IN may contributed to the growing lifetime risk. Antibiotic therapy is the main treatment for UTI, with commonly used antibiotics such as cephalosporins and fluoroquinolones. However, antibiotic resistance is a major challenge in treating UTI. Studies have shown that, 39.0% of levofloxacin and 42% of cephalosporin resistance were found in Asia [18]. Therefore, combining the lifetime risk and bacterial resistance spectrum of UTI in different regions is an effective strategy for preventing and controlling UTI. In high SDI region, medications are implicated to 78% of IN [19, 20]. Antibiotics, proton pump inhibitors, and nonsteroidal anti-inflammatory drugs (NSAIDs) are the mainly inducer of IN [21]. Notably, immune checkpoint inhibitors induced IN (ICI-IN) is increasing due to the its rising use in solid tumor [22], which need to be taken into account when making prevention policies, especially in high SDI regions.

Lifetime risk alterations for UTI and IN across age groups revealed distinct patterns. Sanitary system should focus on early prevention and management strategies for females under 40 and males under 80, as these groups exhibit higher developing risks. Using an ARIMA model, the projected global lifetime risk of developing and dying from UTI and IN over the next 3 decades indicates a persistent upward trend, consistent with historical patterns. However, the widening prediction confidence intervals suggest increasing uncertainty and potential variability in future trends. Despite this, the overall magnitude of change is expected to remain relatively modest.

While this study provides comprehensive estimates of lifetime risk patterns, several limitations warrant consideration. First, the accuracy of lifetime risk calculations depends on the quality of underlying incidence and mortality data, which may be compromised in low-SDI regions due to incomplete vital registration systems. Second, as healthcare conditions and social environments evolve, lifetime risk may change over time. Moreover, it cannot fully reflect the specific risks of individuals or specific regions because of the imbalance development. Lastly, ARIMA model rely on historical trends and may not account for emergent risks in change.

## Conclusion

In conclusion, this study illustrated the lifetime developing and dying rate of UTI and IN in different gender, different region, different social economy, depicting the current popularity of UTI and IN globally, addressing the challenge in controlling the UTI and IN.

## Supplementary Information

Additional file 1.

Additional file 2.

## Data Availability

No datasets were generated or analysed during the current study.
