# Supplementary material for: Global, regional, and national lifetime probabilities of urinary tract infections and interstitial nephritis from 1990 to 2021
Source: J Health Popul Nutr. 2025 Jul 3;44:231. doi: 10.1186/s41043-025-00950-y (PMC12224759; doi:10.1186/s41043-025-00950-y)
Supplement: Supplementary file 1 — Additional file 1. [file 41043_2025_950_MOESM1_ESM.docx]

**Supplementary methods part:**

**Methods of calculating lifetime risk of** **developing and dying from UTI and IN**

1. Cumulative rate

$$Cumulative rate=\sum_{i=1}^{A} W_{i}R_{i}$$

A represents the age band for cumulative rate calculations. Wi denotes the width of the ii-th age interval, and RiRi​ represents the age-specific incidence rate.

1. Cumulative risk

The cumulative risk can be calculated by the formular:

$$Cumulate risk=1-e^{-cumulative rate}$$

1. The “adjusted for multiple primaries (AMP)” method

The AMP method has been introduced in detail in previously study ^1,2^. Lifetime risk was calculated by five-year age groups using all-cause mortality and UTI and IN incidence and mortality data:

$$S=\int_{0}^{\infty} \lambda_{c}\left( a \right)S_{0}\left( a \right)\mathrm{da}=\sum_{i=1}^{f} \frac{R_{i}}{R_{i}+M_{i}-D_{i}}\hat{S}_{0}^{*}\left( a_{i} \right)\times\left( 1-exp\left( -\frac{w_{i}}{N_{i}}\left( R_{i}+M_{i}-D_{i} \right) \right) \right)$$

For age group i:

S: Probability of being diagnosed with UTI and IN;

M_i​_: Annual number of deaths (all-cause mortality);

D_i_​: Annual number of UTI and IN-related deaths;

R_i_​: Annual number of UTI and IN cases;

N_i_​: Population size;

$\lambda_{c}$​: UTI and IN incidence rate;

S ̂_0^* ($a_{i}$): Probability of being alive and UTI and IN-free at age ai;

$W_{i}$: Width of the $i^{th}$ age group;

where the $i^{th}$ interval is from $a_{i}$ to $a_{i+1}$,

$W_{i}$_=_ ($a_{i}-a_{i+1}$)

$$\hat{S}_{0}^{*}\left( a_{i} \right)=exp (-\sum_{j=1}^{i-1} \frac{R_{j}{+ (M}_{j}-D_{j})}{N_{j}})$$

In practice, UTI and IN incidence and mortality data are usually presented in the form of frequency table for calculation, and the final age band is usually 85 years old or above (e.g.0-4,5-4,10-10, …, 80-84, 85+). For the final age band (e.g. 85+), the integral for the final age band evaluates to the following:

$$\frac{R_{f}}{R_{f}+M_{f}-D_{f}}S_{0}\left( a_{f} \right)$$

The derivation of variance to the following:

let： $\lambda_{c}=\frac{R_{i}}{R_{i}+M_{i}-D_{i}},\hat{S}_{0}^{*}=\hat{S}_{0}^{*}\left( a_{i} \right),S_{x}=\left( 1-\exp\left( -\frac{w_{i}}{N_{i}}\left( R_{i}+M_{i}-D_{i} \right) \right) \right)$ then

$Var\left( S \right)=\sum_{i=1}^{f} Var\left( \lambda_{c}\hat{S}_{0}^{*}S_{x} \right)$=$\sum_{i=1}^{f} \left[ E\left( \lambda_{c}^{2}\left( \hat{S}_{0}^{*} \right)^{2}S_{x}^{2} \right)-{E\left( \lambda_{c}\hat{S}_{0}^{*}S_{x} \right)}^{2} \right] (1)$

$$E\left( \lambda_{c}^{2}\left( \hat{S}_{0}^{*} \right)^{2}S_{x}^{2} \right)=E (\lambda_{c}^{2})E\left( \hat{S}_{0}^{*} \right)^{2}E\left( S_{x}^{2} \right)=\left( var\hat{(S}_{0}^{*})+{(E\hat{S}_{0}^{*})}^{2}][Var\left( S_{x} \right)+{E (S_{x})}^{2}]\} \right)$$

$$Var\left( S \right)=\sum_{i=1}^{f} \{[Var (\lambda_{c})+\left( E\lambda_{c} \right)^{2}][Var\left( \hat{S}_{0}^{*} \right)+\left( E\hat{S}_{0}^{*} \right)^{2}][Var\left( S_{x} \right)+\left( ES_{x} \right)^{2}-\left( E\lambda_{c} \right)^{2}\left( E\hat{S}_{0}^{*} \right)^{2}\left( ES_{x} \right)^{2}\}$$

Let:

$p_{ci}=\frac{R_{i}}{R_{i}+M_{i}-D_{i}},$ then

$$E\lambda_{c}=p_{ci}, \left( Binomial\& Poisson \right) (2)$$

$$Var\left( \lambda_{c} \right)=\left\{ \begin{aligned} \frac{p_{ci}\left( 1-p_{ci} \right)}{R_{i}+M_{i}-D_{i}}, \left( Binomial \right) \\ \frac{p_{ci}}{R_{i}+M_{i}-D_{i}}, \left( Poisson \right) \end{aligned} (3) \right.$$

$\log\left( \hat{S}_{0}^{*} \right)=-\sum_{j=1}^{i-1} \frac{R_{j}+M_{j}-D_{j}}{N_{j}}$, let $p_{oj}=\frac{R_{j}+M_{j}-D_{j}}{N_{j}},$ then

$$Var\left( log\hat{S}_{0}^{*} \right)=\sum_{j=1}^{i-1} Var\left( \frac{R_{j}+M_{j}-D_{j}}{N_{j}} \right)=\left\{ \begin{aligned} \sum_{j=1}^{i-1} \frac{p_{oj}\left( 1-p_{oj} \right)}{N_{j}}, (Binomial) \\ \sum_{j=1}^{i-1} \frac{p_{oj}}{N_{j}}, (Poisson) \end{aligned} \right.$$

According to delta method, if $\hat{\theta}\sim N\left( \theta,\sigma^{2} \right)$, then $f\left( \hat{\theta} \right)\sim N (f\left( \theta\right),{[f^{'}\left( \theta\right)]}^{2}\sigma^{2})$. Let $\hat{S}_{0}^{*}=e^{\hat{\theta}}, then$

$$E\left( \hat{S}_{0}^{*} \right)=\exp\left( -\sum_{j=1}^{i-1} p_{oj} \right), \left( Binomial \& Poisson \right) (4)$$

$$Var\left( \hat{S}_{0}^{*} \right)={(e^{\hat{\theta}})}^{2}Var\left( log\hat{S}_{0}^{*} \right)=\left( \hat{S}_{0}^{*} \right)^{2}Var\left( log\hat{S}_{0}^{*} \right)=\left\{ \begin{aligned} \left( \hat{S}_{0}^{*} \right)^{2}\times\sum_{j=1}^{i-1} \frac{p_{oj}\left( 1-p_{oj} \right)}{N_{j}}, \left( Binomial \right) \\ \left( \hat{S}_{0}^{*} \right)^{2}\times\sum_{j=1}^{i-1} \frac{p_{oj}}{N_{j}}, \left( Poisson \right) \end{aligned} \right. (5)$$

$S_{x}=\left( 1-\exp\left( -\frac{w_{i}}{N_{i}}\left( R_{i}+M_{i}-D_{i} \right) \right) \right)$,

Let $S_{x}^{'}=\exp\left( -\frac{w_{i}}{N_{i}}\left( R_{i}+M_{i}-D_{i} \right) \right),$ $\log\left( S_{x}^{'} \right)=-\frac{w_{i}}{N_{i}}\left( R_{i}+M_{i}-D_{i} \right)$, and $p_{xi}=\frac{R_{i}}{R_{i}+M_{i}-D_{i}}$，then

Var ($\log S_{x}^{'})=\left\{ \begin{aligned} {w_{i}}^{2}\frac{p_{xi}\left( 1-p_{xi} \right)}{N_{i}}, (Binomial) \\ {w_{i}}^{2}\frac{p_{xi}}{N_{i}}, (Poisson) \end{aligned} \right.$

Let $S_{x}=1-e^{\hat{\theta}},$ according to delta method，then：

$$E\left( S_{x} \right)=\left( 1-\exp\left( -\frac{w_{i}}{N_{i}}\left( R_{i}+M_{i}-D_{i} \right) \right) \right), \left( Binomial \& Poisson \right) (6)$$

$Var\left( S_{x} \right)={(e^{\hat{\theta}})}^{2}\times Var\left( e^{\hat{\theta}} \right)=\left\{ \begin{aligned} {(S_{x}^{'})}^{2}\times{w_{i}}^{2}\frac{p_{xi}\left( 1-p_{xi} \right)}{N_{i}}, (Binomial) \\ {(S_{x}^{'})}^{2}\times{w_{i}}^{2}\frac{p_{xi}}{N_{i}}, (Poisson) \end{aligned} \right.$ (7)

**Frontier Analysis**

To assess the relationship between the burden of urinary tract infections (UTI) and interstitial nephritis (IN) and socio-demographic development, we employed “Frontier Analysis” as a quantitative method to identify the minimum achievable lifetime incidence and mortality risks based on developmental status measured by the “Socio-demographic Index (SDI)”. Frontier Analysis determines the lowest attainable lifetime risk level for each country or region according to its SDI. The deviation from the frontier, termed the “effective difference”, reflects unachieved improvement opportunities (i.e., reductions in lifetime of UTI and IN) that could theoretically be realized given the country’s position on the development spectrum.

We applied Data Envelopment Analysis (DEA) using the Free Disposal Hull (FDH) approach to construct a nonlinear frontier, generating UTI and IN frontiers stratified by SDI from 1990 to 2021 ^3,4^. To account for uncertainty, 1,000 bootstrap samples were drawn via random resampling (with replacement) across all countries and years. The average lifetime risk of UTI and IN was calculated for each SDI value. A locally weighted regression (LOESS) with a local polynomial degree of 1 and a span of 0.2 was then applied to generate a smoothed frontier curve. To mitigate outlier effects, super-efficient countries were excluded during frontier construction.

This methodology enables the identification of optimal risk reduction targets tailored to regional development levels, informing context-specific public health strategies to mitigate UTI and IN burdens globally ^3^.

**Concentration Index**

The Concentration Index is introduced by Kakwani^5^ and Wagstaff et al.^6^, the Concentration Index is define as^7^:

$$E\left( h \right)=\frac{8}{n^{2}\left( a_{h}-b_{h} \right)}\sum_{i=1}^{n} z_{i}h_{i}$$

$E\left( h \right)$ is the only rank-dependent socioeconomic indicator which satisfies the four properties and has maximum bounds equal to -1 and +1. The actual bounds between which the indicator varies depend upon the mean health level $\mu_{h}$. It can be shown quite easily that:

$$-\frac{4\left( b_{h}-u_{h} \right)\left( u_{h}-a_{h} \right)}{\left( b_{h}-a_{h} \right)^{2}}\leq E\left( h \right)\leq+\frac{4\left( b_{h}-\mu_{h} \right)\left( \mu_{h}-a_{h} \right)}{\left( b_{h}-a_{h} \right)^{2}}$$

The bounds tend to zero if $\mu_{h}\to a_{h}$. They increase if the mean rises, and reach a maximum when uh is exactly halfway between $a_{h}$ and $b_{h}$, at which point the bounds are equal to -1 and +1. (when $n$ is even and there exist no persons who are neither rich nor poor; otherwise, the bounds are slightly smaller in absolute value). Then they decrease, and tend to zero again if $\mu_{h}\to b_{h}$.

**ARIMA model**

The ARIMA model, conventionally denoted as ARIMA(p,d,q), incorporates three core parameters: p indicating autoregressive order, d representing differencing degree, and q specifying moving average order ^8^. This comprehensive modeling system encompasses various fundamental components including ARMA configurations along with elementary autoregressive (AR), integrated (I), and moving average (MA) submodels. In temporal sequence analysis, the AR(p) formulation establishes current observation Yt through linear combinations of p historical values $Y_{t-1}, Y_{t-2},\cdots,Y_{t-p}$ and the current residuals εt. MA (q) model refers to the current value of the time series $Y_{t}$ linearly in terms of its current and previous residual series $\varepsilon_{t-1},\varepsilon_{t-2},\cdots\varepsilon_{t-q}$.The general formula of AR (p) and MA (q) models can be expressed as follow, respectively.

$$Y_{t}=\phi_{1}Y_{t-1}+\phi_{2}Y_{t-2}+\cdots\phi_{P}Y_{t-P}+\varepsilon_{t}$$

$$Y_{t}=\theta_{1}\varepsilon_{t-1}-\theta_{2}\varepsilon_{t-2}-\cdots\theta_{q}\varepsilon_{t-q}-\varepsilon_{t}$$

where $\phi$ and $\theta$ are the autoregressive and moving average parameters, respectively. Yt is the observed value at time t and εt is the value of the random shock at time t. It is assumed to be independently and identically distributed with a mean of zero and a constant variance of $\sigma^{2}$. ARMA(p,q) model is composed of AR and MA models, in which the current value of the time series is defined linearly in terms of its previous values as well as current and previous residual series. The ARMA(p,q) model can be presented as given:

$$Y_{t}=\alpha+\phi_{1}Y_{t-1}+\phi_{2}Y_{t-2}+\cdots\phi_{P}Y_{t-P}+\varepsilon_{t}-\theta_{1}\varepsilon_{t-1}-\theta_{2}\varepsilon_{t-2}-\cdots-\theta_{q}\varepsilon_{t-q}$$

Where $\alpha$ is a constant, $\varepsilon_{t-1}$ is the value of the previous random shock. The ARIMA model deals with non-stationary time series. The differenced stationary time series can be modelled as an ARMA model to perform the ARIMA model ^9^.

**References**:

1 Sasieni, P. D., Shelton, J., Ormiston-Smith, N., Thomson, C. S. & Silcocks, P. B. What is the lifetime risk of developing cancer?: the effect of adjusting for multiple primaries. *British journal of cancer* **105**, 460-465, doi:10.1038/bjc.2011.250 (2011).

2 Ahmad, A. S., Ormiston-Smith, N. & Sasieni, P. D. Trends in the lifetime risk of developing cancer in Great Britain: comparison of risk for those born from 1930 to 1960. *British journal of cancer* **112**, 943-947, doi:10.1038/bjc.2014.606 (2015).

3 Healthcare Access and Quality Index based on mortality from causes amenable to personal health care in 195 countries and territories, 1990-2015: a novel analysis from the Global Burden of Disease Study 2015. *Lancet (London, England)* **390**, 231-266, doi:10.1016/s0140-6736(17)30818-8 (2017).

4 Xie, Y., Bowe, B., Xian, H., Balasubramanian, S. & Al-Aly, Z. Rate of Kidney Function Decline and Risk of Hospitalizations in Stage 3A CKD. *Clinical journal of the American Society of Nephrology : CJASN* **10**, 1946-1955, doi:10.2215/cjn.04480415 (2015).

5 Kakwani, N. C. *Income inequality and poverty*. (World Bank New York, 1980).

6 Wagstaff, A., Paci, P. & van Doorslaer, E. On the measurement of inequalities in health. *Social Science & Medicine* **33**, 545-557, doi:<https://doi.org/10.1016/0277-9536(91)90212-U> (1991).

7 Erreygers, G. Correcting the concentration index. *Journal of health economics* **28**, 504-515, doi:10.1016/j.jhealeco.2008.02.003 (2009).

8 Li, X., Zhang, C., Zhang, B. & Liu, K. A comparative time series analysis and modeling of aerosols in the contiguous United States and China. *The Science of the total environment* **690**, 799-811, doi:10.1016/j.scitotenv.2019.07.072 (2019).

9 He, Z. & Tao, H. Epidemiology and ARIMA model of positive-rate of influenza viruses among children in Wuhan, China: A nine-year retrospective study. *International journal of infectious diseases : IJID : official publication of the International Society for Infectious Diseases* **74**, 61-70, doi:10.1016/j.ijid.2018.07.003 (2018).

**Supplementary figures**:


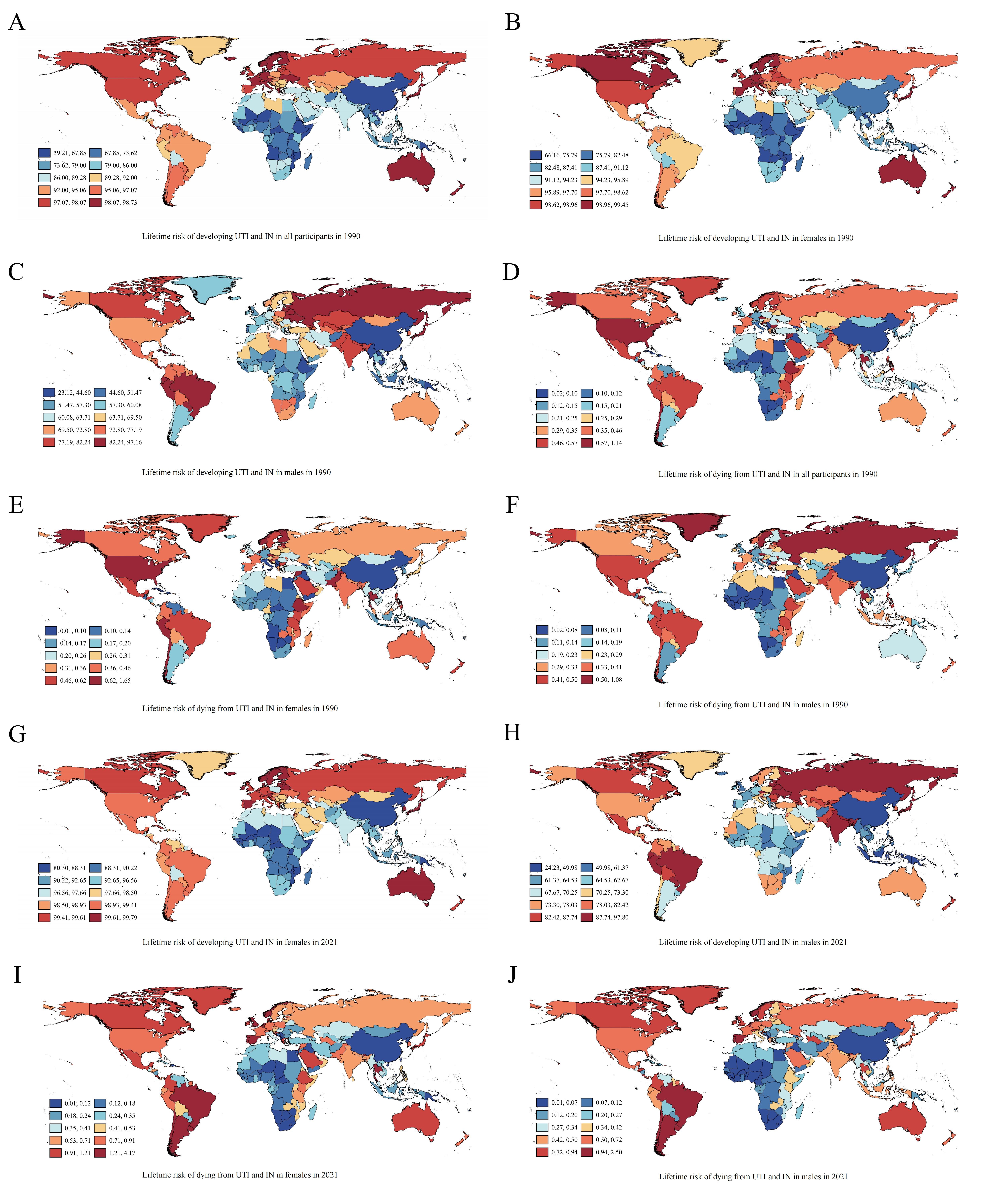


**Figure S1. Lifetime risk of developing and dying from UTI and IN across 204 countries in 1990 and 2021 globally.** (A-C) Lifetime risk of developing UTI and IN in all participants (A), female (B) and male (C) in 1990. (D-F) Lifetime risk of dying from UTI and IN in all participants (D), female (E) and male (F) in 1990. (G, H) Lifetime risk of developing UTI and IN in female (G) and male (H) in 2021. (I, J) Lifetime risk of developing UTI and IN in female (I) and male (J) in 2021.


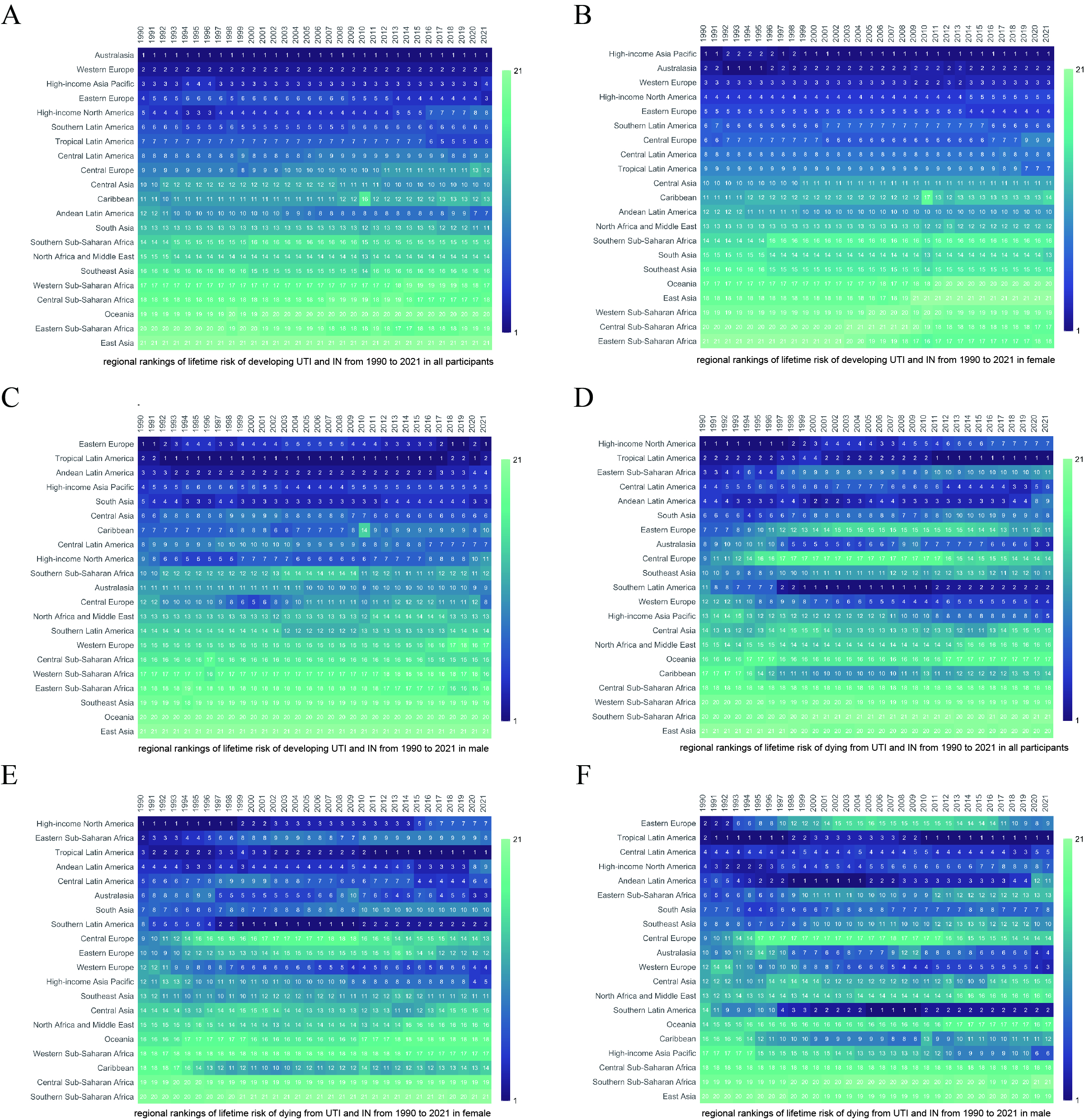


**Figure S2. Regional ranking in lifetime risk of developing and dying from UTI and IN from 1990 to 2021 displayed by heatmap.** (A-C) Lifetime risk of developing and dying from UTI and IN in all participants.

**
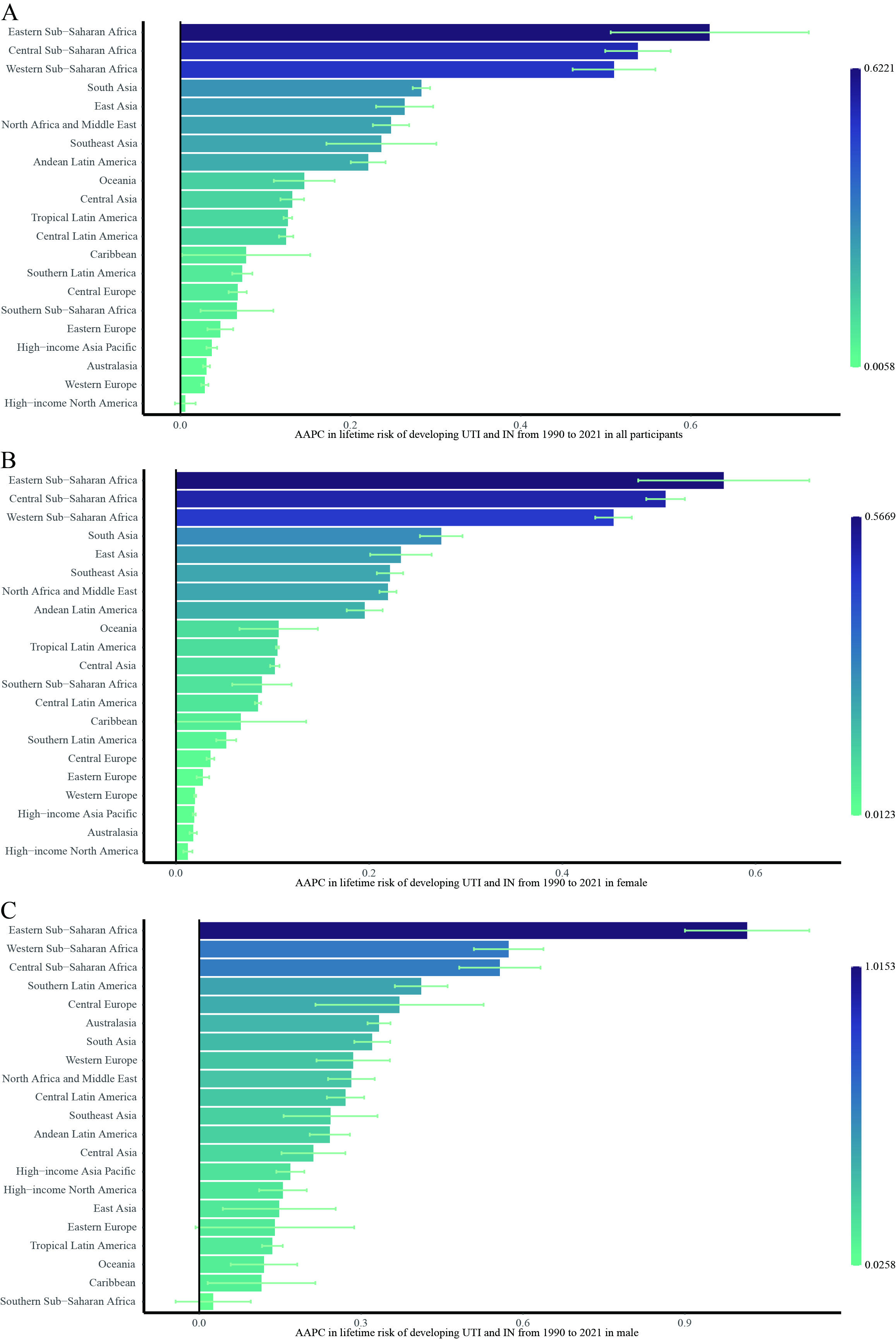
**

**Figure S3. AAPC in lifetime risk of developing UTI and IN from 1990 to 2021.** (A) all participants, (B) female and (C) male respectively. The legend on the right indicated the magnitude of AAPC. AAPC greater than 0 signified an increasing trend in lifetime risk, while an AAPC less than 0 indicates a decreasing trend.


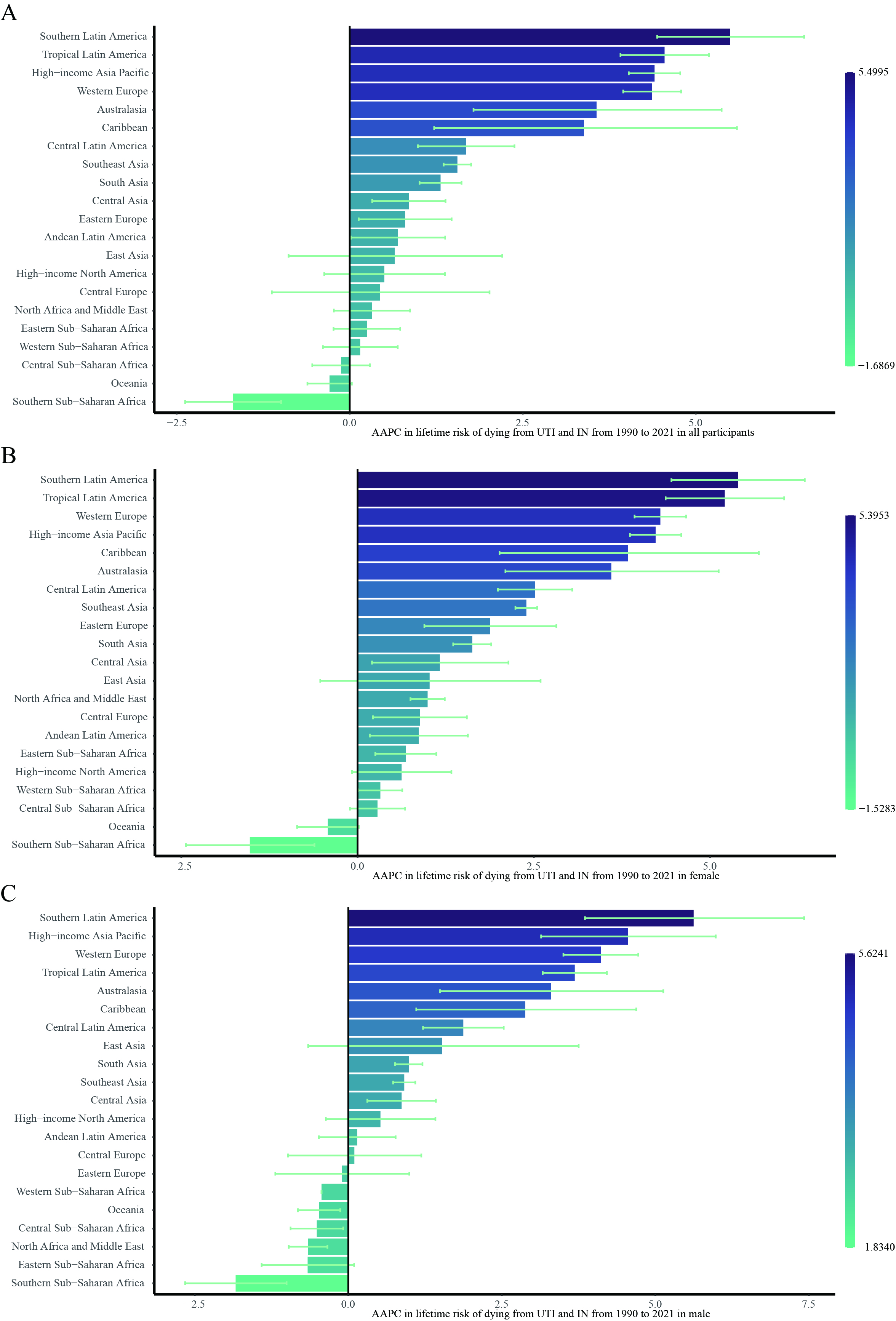


**Figure S4. AAPC in lifetime risk of dying from UTI and IN from 1990 to 2021.** (A) all participants, (B) female and (C) male respectively. The legend on the right indicated the magnitude of AAPC. AAPC greater than 0 signified an increasing trend in lifetime risk, while an AAPC less than 0 indicates a decreasing trend.

**
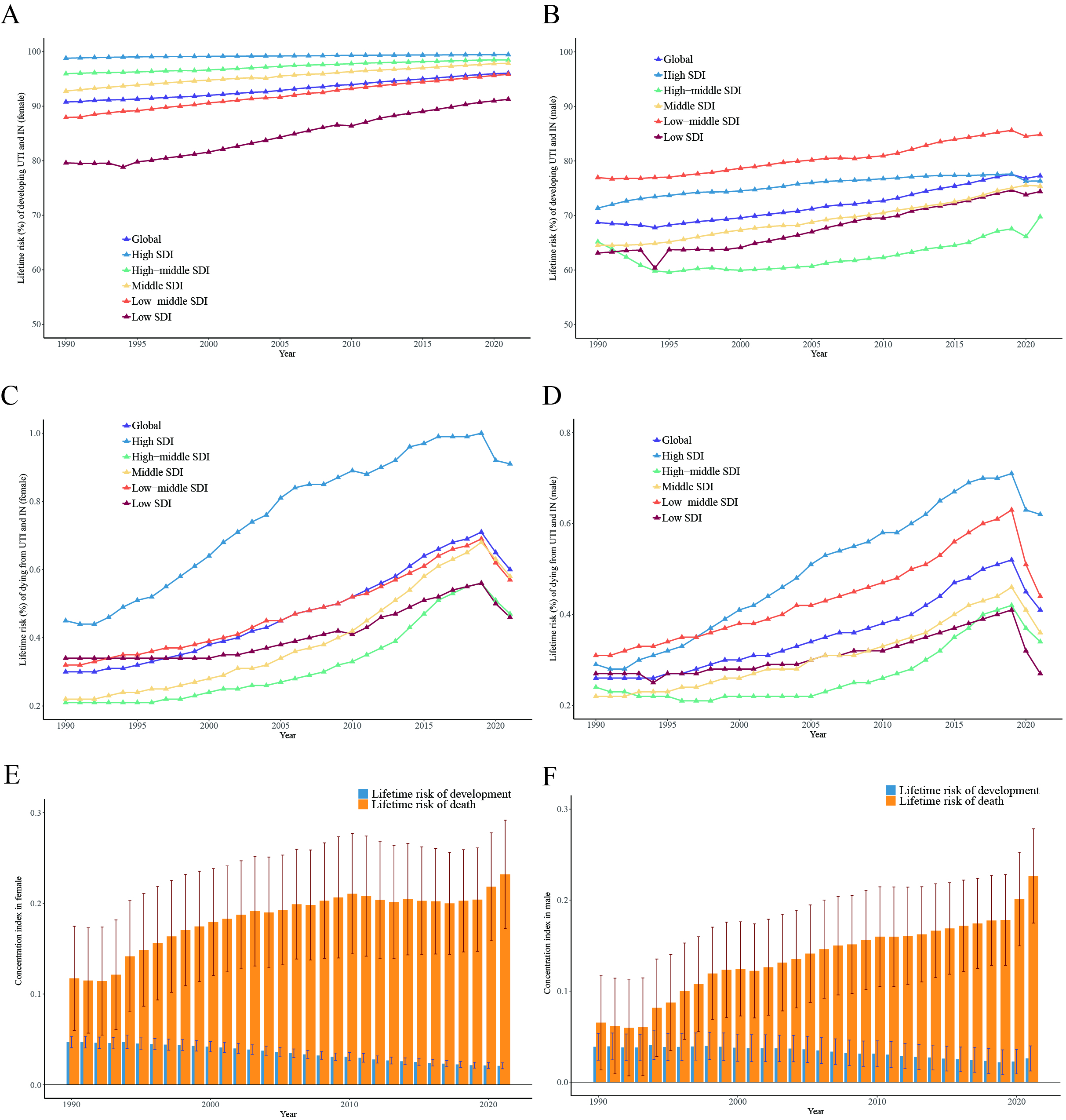
**

**Figure S5. Relationship between socioeconomic inequality and lifetime risk of UTI and IN.** (A, B) Lifetime risk of developing UTI and IN in different SDI levels from 1990 to 2021 in female (A) and male (B). (C, D) Lifetime risk of dying from UTI and IN in different SDI levels from 1990 to 2021 in female (C) and male (D). (E, F) Concentration index values of developing and dying lifetime risk from 1990 to 2021 globally in female(E) and male (F). The error bars denote the 95% CI.


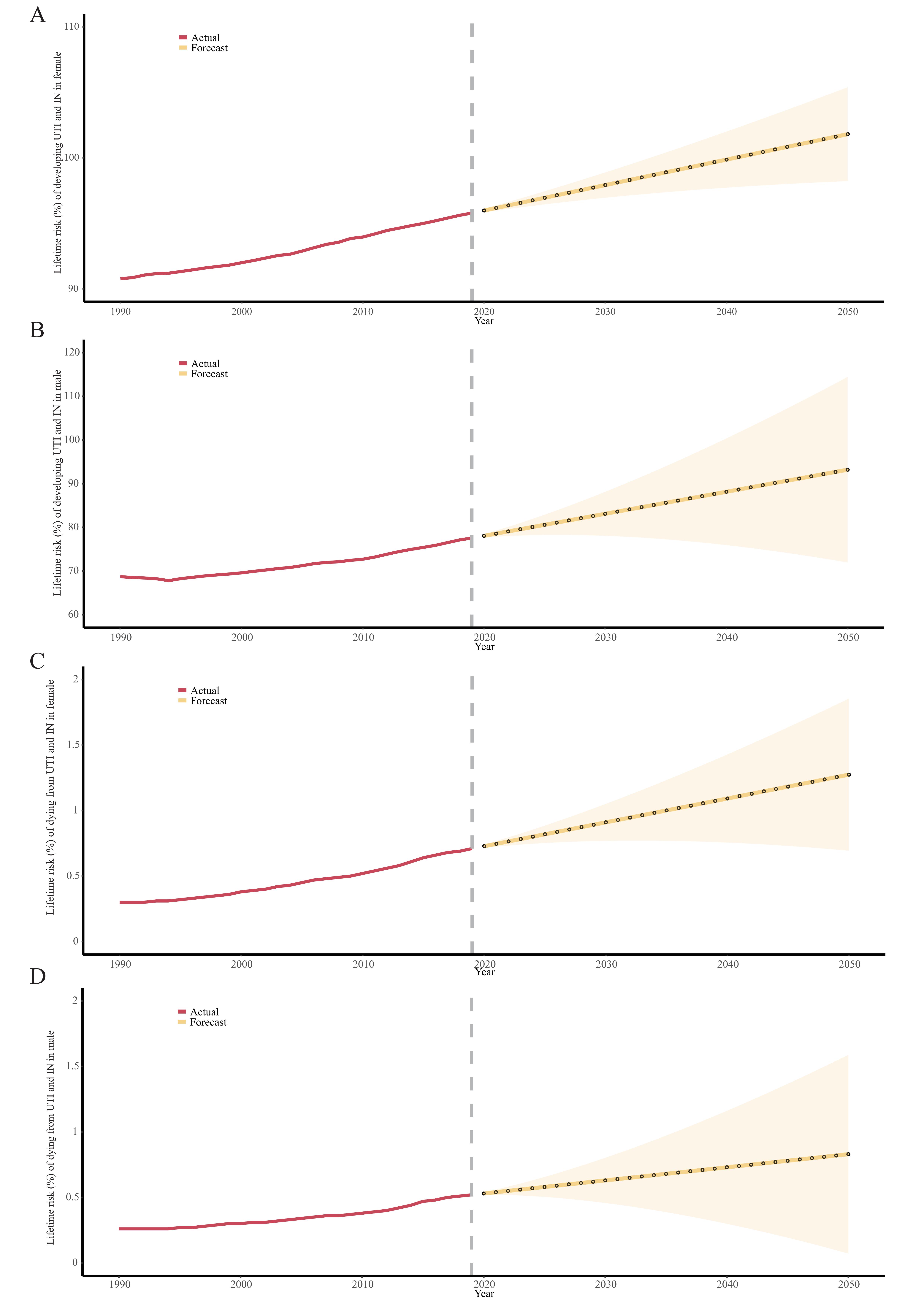


**Figure S6. ARIMA model forecasts lifetime risk of developing UTI and IN in the next 30 years concerning SDI level.** (A, B) Curves of forecasted lifetime risk of developing UTI and IN in female (A) and male (B) in the next 30 years. (C, D) Curves of forecasted lifetime risk of dying from UTI and IN in female (C) and male (D) in the next 30 years. The red curve represented the lifetime risk of developing (or dying risk) UTI and IN from 1990 to 2019 and the yellow curve forecasted the developing risk from 2020 to 2050. Yellow regions represented 95% CI for the forecasted risk.
